# Protein O-GlcNAc Modification Increases in White Blood Cells After a Single Bout of Physical Exercise

**DOI:** 10.3389/fimmu.2018.00970

**Published:** 2018-05-03

**Authors:** Tamás Nagy, Emese Kátai, Viktória Fisi, Tamás Tibor Takács, Antal Stréda, István Wittmann, Attila Miseta

**Affiliations:** ^1^Department of Laboratory Medicine, Medical School, University of Pécs, Pécs, Hungary; ^2^János Szentágothai Research Centre, University of Pécs, Pécs, Hungary; ^3^Department of Internal Medicine and Nephrology Center, Medical School, University of Pécs, Pécs, Hungary

**Keywords:** exercise, O-GlcNAc, stress response, white blood cells, exercise immunology

## Abstract

**Background:**

Protein O-linked *N*-acetylglucosamine (O-GlcNAc) is a dynamic posttranslational modification influencing the function of many intracellular proteins. Recently it was revealed that O-GlcNAc regulation is modified under various stress states, including ischemia and oxidative stress. Aside from a few contradictory studies based on animal models, the effect of exercise on O-GlcNAc is unexplored.

**Purpose:**

To evaluate O-GlcNAc levels in white blood cells (WBC) of human volunteers following physical exercise.

**Methods:**

Young (age 30 ± 5.2), healthy male volunteers (*n* = 6) were enlisted for the study. Blood parameters including metabolites, ions, “necro”-enzymes, and cell counts were measured before and after a single bout of exercise (2-mile run). From WBC samples, we performed western blots to detect O-GlcNAc modified proteins. The distribution of O-GlcNAc in WBC subpopulations was assessed by flow cytometry.

**Results:**

Elevation of serum lactic acid (increased from 1.3 ± 0.4 to 6.9 ± 1.7 mM), creatinine (from 77.5 ± 6.3 U/L to 102.2 ± 7.0 μM), and lactate dehydrogenase (from 318.5 ± 26.2 to 380.5 ± 33.2 U/L) confirmed the effect of exercise. WBC count also significantly increased (from 6.6 ± 1.0 to 8.4 ± 1.4 G/L). The level of O-GlcNAc modified proteins in WBCs showed significant elevation after exercise (85 ± 51%, *p* < 0.05). Flow cytometry revealed that most of this change could be attributed to lymphocytes and monocytes.

**Conclusion:**

Our results indicate that short-term exercise impacts the O-GlcNAc status of WBCs. O-GlcNAc modification could be a natural process by which physical activity modulates the immune system. Further research could elucidate the role of O-GlcNAc during exercise and validate O-GlcNAc as a biomarker for fitness assessment.

## Introduction

Physical exercise is generally acknowledged as a beneficial activity and widely promoted to increase physical fitness, the quality of life, and life expectancy (1–4). In fact, exercise (especially regular exercise) not exceeding our actual tolerance level is a way to precondition and protect our body from future harmful events (5, 6). During physical exercise, the cells in our body have to adapt to a significantly more challenging condition than during regular, resting state. Mechanical workload and strain increases in the musculoskeletal system, hemodynamic shear stress is intensified on vascular and circulating cells, largely enhanced metabolism in the muscles generates free radicals and acidosis while other tissues’ metabolism has to temporarily decline (7–11). Overall, exercise is a type of stress and the cells has to adapt to it by various stress response mechanisms (5, 12). The underlying molecular events of the stress response elicited are numerous, including regulation of mitochondrial activity, increased expression of heat-shock proteins, and elements of the free radical scavenge system (13–16). Although intensively studied, the understanding of how these processes are activated is still not complete.

In recent years, protein O-linked *N*-acetylglucosamine (O-GlcNAc) modification emerged as a possible signaling mechanism that could mediate the effects of intracellular metabolic and stress response (17). O-GlcNAc is a dynamic and reversible posttranslational sugar modification on serine and threonine residues of intracellular proteins by attachment or removal of a single *N*-acetylglucosamine molecule. O-GlcNAc modification is similar to phosphorylation; indeed, it may compete for the same Ser/Thr sites with phosphorylation, although other types of interplays, such as proximal site competition and proximal site occupation were also proposed (18). As detection methods improve, the number of proteins known to be O-GlcNAc modified grows significantly; around 1,500 proteins were identified to be influenced by O-GlcNAc (19, 20), including signaling components, transcriptional factors, and metabolic enzymes. An important feature of O-GlcNAc modification is that it closely connected to carbohydrate metabolism. The substrate for O-GlcNAc modification is uridine diphosphate *N*-acetylglucosamine (UDP-GlcNAc), the end product of the hexosamine biosynthesis pathway which depends on the availability of glucose in the cells. On the other hand, O-GlcNAc was found to act as a nutrient sensor, regulating the glucose uptake and insulin resistance of the cells (21). Interestingly, Zachara et al. showed in their 2004 paper that stress also induced an increase of O-GlcNAc modification (22). Since then, many study demonstrated that O-GlcNAc is sensitive for a variety of stressors, including hypoxia and oxidative stress. Moreover, accumulating data—mostly collected in studies using cardiomyocytes or cardiac animal models—suggest that elevated O-GlcNAc has a protective role in hypoxia and/or oxidative stress related injuries (23).

O-GlcNAc has been also proposed as a mediator element in preconditioning experiments, e.g., Vibjerg Jensen et al. found that two 5 min long no-flow ischemia in isolated perfused rat hearts elevated O-GlcNAc levels and argue that this process might contribute to the cardioprotection (24). Jones et al. had similar results and also concluded that O-GlcNAc may protect by modifying mitochondrial proteins such as voltage-dependent anion channels (25). The effect of physical exercise on O-GlcNAc levels were studied in only a limited number of animal models, with controversial results. Long-term (regular exercise) effects were found to either increase or decrease O-GlcNAc levels in rodent cardiac and muscle tissues (26–29). There are only two studies available that analyzed the effect of acute exercise on protein O-GlcNAc modification. Peternelj et al. showed increased O-GlcNAc levels in rat skeletal muscle following acute exercise (running until exhaustion) (30). By contrast, Medford et al. found that O-GlcNAc levels decreased after 15 min but did not change significantly after 30 min of treadmill running in mice hearts (31). Although no study was conducted, yet on blood samples following acute exercise, analysis of leukocytes and leukocyte-derived cell lines suggest that leukocytes can and will respond with altered O-GlcNAc levels to activation and to metabolic challenges (32–35). Moreover, it was also suggested that changing O-GlcNAc levels may have immunomodulatory effects (36). Since the immune system is deeply involved in our body’s response to physical exercise, it would be of significant value to clarify the possible role of O-GlcNAc in exercised induced limited inflammatory response (37).

In our present study, we hypothesized that physical exercise would elicit a limited stress response of the body that would include altered O-GlcNAc protein modification and that this process would manifest at least in some elements of the cellular immunity. Thus, our aim was to investigate whether O-GlcNAc levels are influenced by a single bout of physical exercise in isolated white blood cells (WBC) of healthy, male humans. Our data reveal that O-GlcNAc modification is a dynamic intracellular process in leukocytes. We also show that subpopulations of leukocytes may have different responses to acute exercise, concerning their O-GlcNAc levels.

## Materials and Methods

### Subjects and Experimental Design

Six male volunteers were recruited for this pilot study, the age of the volunteers ranged from 24 to 39 years. All subjects were informed about the procedures and the risks of the experiments before obtaining written informed consents. All procedures were approved by the Regional Committee for Research Ethics of the University of Pécs, Hungary (approval No.: 5187). The selection criteria for the participants included no obesity, no smoking, no regular medications taken, and no known acute or chronic disease present (Table 1). The experimental setup was designed to contain two (almost identical) phases: (1) resting and (2) exercise. The two phases were separated by a 3-week period. In each phases, the participants were asked to report at the laboratory in the morning after 12 h of fasting and 48 h of restrain from any strenuous physical exercise. Venous blood was drawn from the cubital veins during both phases (before resting and before exercise samples). Next, the volunteers received identical breakfasts (~600 kcal, 80% carbohydrate). In the first phase, the volunteers were asked after breakfast to restrain from any physical activity for 3.5 h before collecting the second set of blood samples (after resting samples). In the second phase, the volunteers were asked 3 h after breakfast to complete a 2-mile running exercise which was followed by the final blood sample collection (after exercise samples). The 2-mile running exercise were carried out and assessed according to the instructions of the US Army physical fitness test (38). Briefly, the participants were asked to complete the 2-mile course in the shortest time possible, without stopping or any physical help.

**Table 1:** Characteristics of the study subjects.

Variable	*n* = 6
Age (years)	30 ± 5.2
Body weight (kg)	77 ± 8.6
BMI (kg/m^2^)	23.94 ± 2.3
History of smoking	0 (0%)
Chronic diseases	0 (0%)
Regular medications	0 (0%)
2-Mile run (duration)	940 ± 85 s
Regular exercise/week (duration)	119 ± 88 min

*Data are mean ± SD*.

### Blood Sampling

Venous blood samples were collected in suitable vacutainers; tubes containing potassium ethylenediaminetetra-acetic acid (K-EDTA) were used for testing cellular blood parameters and to isolate WBCs. Tubes containing sodium-fluoride (NaF) as glycolysis inhibitor were used for plasma glucose and lactate analysis, while tubes without additives were used to obtain serum for the routine laboratory blood tests (Table 2.). After blood collection, plasma and serum were separated by centrifugation (10 min, room temperature, 1,500 rcf). Blood cell parameters were quantified in a multi-parameter automatic hematology analyzer Cell-Dyn 3700 system (Abbott Diagnostics, Abbott Laboratories, Abbott Park, IL, USA). Plasma and serum parameters were measured by Cobas 8000 Modular Analyzer (Roche Diagnostics, GmbH, Mannheim, Germany) following the manufacturer’s instructions.

**Table 2:** Serum biochemical and blood cell parameters before and after exercise or resting.

	Resting		Exercise		
	Before	After	Before	After	Units
Sodium	140.7 ± 1.1	140.2 ± 1.1	141.4 ± 1.2	142.0 ± 1.3	mM
Potassium	4.3 ± 0.3	4.6 ± 0.2	4.3 ± 0.2	4.2 ± 0.2	mM
Calcium	2.4 ± 0.0	2.5 ± 0.1	2.4 ± 0.1	2.5 ± 0.1^‡^	mM
Magnesium	0.9 ± 0.1	0.9 ± 0.0	0.9 ± 0.1	0.9 ± 0.1	mM
Chloride	97.8 ± 1.6	98.2 ± 1.3	101.6 ± 2.8*	99.8 ± 2.7	mM
Phosphate	1.2 ± 0.1	1.2 ± 0.1	1.1 ± 0.2	1.5 ± 0.3^*,#,‡^	mM
Insulin	40.0 ± 8.0	59.5 ± 9.3*	54.0 ± 29.6	51.9 ± 38.9	pM
Lactate	1.4 ± 0.5	1.4 ± 0.4	1.3 ± 0.4	6.9 ± 1.7^*,#,‡^	mM
Glucose	5.2 ± 0.3	4.8 ± 0.7	5.1 ± 0.4	6.4 ± 1.6	mM
Bilirubin	13.7 ± 6.7	13.5 ± 7.2	14.8 ± 7.8	13.7 ± 7.8	μM
Urea	5.6 ± 1.6	5.2 ± 1.4	5.4 ± 1.3	5.2 ± 1.1	mM
Creatinine	86.5 ± 9.6	78.2 ± 9.3*	77.5 ± 6.3	102.2 ± 7.0^*,#,‡^	μM
Uric acid	319.7 ± 53.5	302.7 ± 59.6	314.2 ± 40.8	355.0 ± 47.4	μM
Cholesterol	4.7 ± 0.7	4.9 ± 0.7	4.7 ± 0.7	4.8 ± 0.7	mM
Triglyceride	1.2 ± 0.4	1.9 ± 0.4*	1.3 ± 0.3	1.5 ± 0.5	mM
Lactate dehydrogenase	318.0 ± 32.9	336.5 ± 56.9	318.5 ± 26.2	380.5 ± 33.2^*,‡^	U/L
Alkaline phosphatase	63.2 ± 10.6	67.0 ± 11.7	74.1 ± 15.4	77.0 ± 13.0	U/L
CK	179.3 ± 81.0	183.8 ± 75.1	196.5 ± 82.3	229.8 ± 90.6	U/L
Total protein	74.0 ± 2.2	76.3 ± 2.6	75.7 ± 4.0	78.0 ± 2.7	g/L
Albumin	48.8 ± 2.0	51.0 ± 1.9*	49.4 ± 2.9	51.9 ± 2.3^*,‡^	g/L
CRP	0.7 ± 0.3	0.7 ± 0.3	0.5 ± 0.2	0.6 ± 0.3	mg/L
White blood cells	6.3 ± 0.8	6.4 ± 1.1	6.6 ± 1.0	8.4 ± 1.4^*,#,‡^	G/L
Neutrophils	3.0 ± 0.7	3.3 ± 1.0	3.2 ± 0.8	3.9 ± 0.9	G/L
Lymphocytes	2.3 ± 0.3	2.2 ± 0.3	2.5 ± 0.3	3.6 ± 0.7^*,#,‡^	G/L
Monocytes	0.4 ± 0.1	0.4 ± 0.0	0.4 ± 0.1	0.5 ± 0.1	G/L
Eosinophils	0.3 ± 0.3	0.2 ± 0.3	0.3 ± 0.3	0.2 ± 0.3	G/L
Basophils	<0.1 ± 0.0	<0.1 ± 0.0	<0.1 ± 0.0	<0.1 ± 0.0	G/L
RBC	5.4 ± 0.2	5.4 ± 0.2	5.2 ± 0.3	5.3 ± 0.3	T/L
Hgb	154.3 ± 7.4	155.5 ± 10.7	152.0 ± 9.4	155.9 ± 9.2	g/L
Hct	47.1 ± 2.0	46.9 ± 2.7	46.9 ± 2.6	46.3 ± 2.2	%
PLT	220.8 ± 17.5	236.8 ± 24.1	233.2 ± 19.3	273.7 ± 30.5^*,#,‡^	G/L

*Data are mean ± SD (*n* = 6)*.

*p < 0.05 vs resting-before.

*^#^p < 0.05 vs resting-after*.

*^‡^p < 0.05 vs exercise-before*.

### Western Blot Analysis

Approximately 2.5 mL of K-EDTA anti-coagulated whole blood was used to isolate mononuclear cells. The anti-coagulated blood samples were layered on Histopaque-1077 (Sigma-Aldrich, St. Louis, MO, USA, Cat. No.: 10771) solution and prepared by isopycnic centrifugation (20 min, RT, 500 rcf) immediately after collection. Mononuclear cells were collected from the plasma/1077 interface and washed 2× in ice-cold PBS. Next, the cells were lysed in a modified RIPA buffer [10 mM Tris pH 7.2, 100 mM NaCl, 1 mM EDTA, 1 mM ethylene glycol-bis(2-aminoethylether)-*N*,*N*,*N*′,*N*′-tetraacetic acid, 0,1% SDS, 1% Triton-X 100, 0.5% deoxycholate, 10% glycerol, protease inhibitor cocktail: 1 tablet/10 mL (Roche Applied Science, Penzberg, Germany)], kept on ice for 30 min and centrifuged for 10 min at 4°C at 14,000 rcf. From the supernatant, the total protein concentration was determined using Bio-Rad *Dc* Assay Kit (Bio-Rad, Hercules, CA, USA). Proteins were separated on 8% SDS-PAGE and transferred onto polyvinylidene difluoride membranes (Millipore, Billerica, MA, USA). Blots were probed with the anti-O-GlcNAc antibody RL-2 (1:1,000; Thermo Fisher Scientific, Waltham, MA, USA, Cat. No.: MA1-072) in 5% non-fat dry milk blocking buffer and followed by HRP conjugated goat anti-mouse IgG (1:5,000; Thermo Fisher Scientific). For loading control, anti-actin IgG antibody (1:1,500, Sigma-Aldrich, Cat. No.: A2103) was used. The blots were developed using Femto chemiluminescent substrate (Thermo Fisher Scientific) and the signal was detected by G:BOX Chemi HR1.4 gel imaging system (Syngene, Cambridge, UK). Densitometry was quantified by calculating average pixel intensities of whole lanes followed by background subtraction using ImageJ analysis software. The O-GlcNAc levels of the samples were normalized for protein content by anti-actin staining.

### Flow Cytometry

Immediately after collection, whole blood samples were treated using Lyse/Fix Buffer (BD Biosciences, Cat. No.: 558049), which simultaneously lysed red blood cells and fixed WBC, according to the manufacturer’s instructions. After discarding the supernatant containing the hemolyzed red blood cells, fixed WBCs were washed once in PBS, and permeabilized with 0.5% Triton X-100-PBS for 2 min. Next, non-specific binding sites were blocked by 5% BSA-PBS for 5 min, and then the cells were incubated with a 1:200 dilution of the anti-O-GlcNAc antibody RL2 in 5% BSA-PBS for 30 min at 37°C. After being rinsed in PBS, the cells were incubated with a 1:200 dilution of secondary antibody fluorescein-conjugated goat anti-mouse IgG (Thermo Fisher Scientific) in 5% BSA-PBS for 30 min at 37°C. Finally, the cells were washed once and then resuspended in PBS before analysis. Forward, side scatter values and fluorescence intensities [detected at 525 nm (FL1 channel)] per cells were measured with a Cytomics FC 500 flow cytometer (Beckman Coulter, Fullerton, CA, USA). Defining the regions of various WBC subpopulations and quantification of data were performed by using FlowJo analysis software.

### Data Analysis

Data are presented as means ± SDs throughout. Comparisons were performed using Student’s *t*-test and statistically significant differences between groups were defined as *p* values <0.05 and are indicated in the legends to the figures. When mentioned, not significant values were indicated as not significant (NS). For serum biochemical and blood cell parameters, two-way repeated measures ANOVA was used to evaluate changes. When significant changes were identified, pairwise comparison with Bonferroni’s correction was used. The type I error rate (α) was set to 0.05.

## Results

### Clinical Characteristic of the Subjects

In this pilot study, six male volunteers participated, their mean age was 30 years (Table 1). All of the participants were considered healthy, with no history of chronic diseases. Smoking or taking medication regularly was not reported. None of the volunteers were obese, and only two of them exceeded the 25 kg/m^2^ BMI mark (26.03 and 27.76). All participants claimed to regularly perform moderate to high intensity exercises, with a minimum duration of 30 min/week. The volunteers performed above the required minimum of their corresponding age group in the 2-mile running exercise, the duration of the exercise ranged from 12:53 to 17:24 min (Table 1).

**Figure 1:**
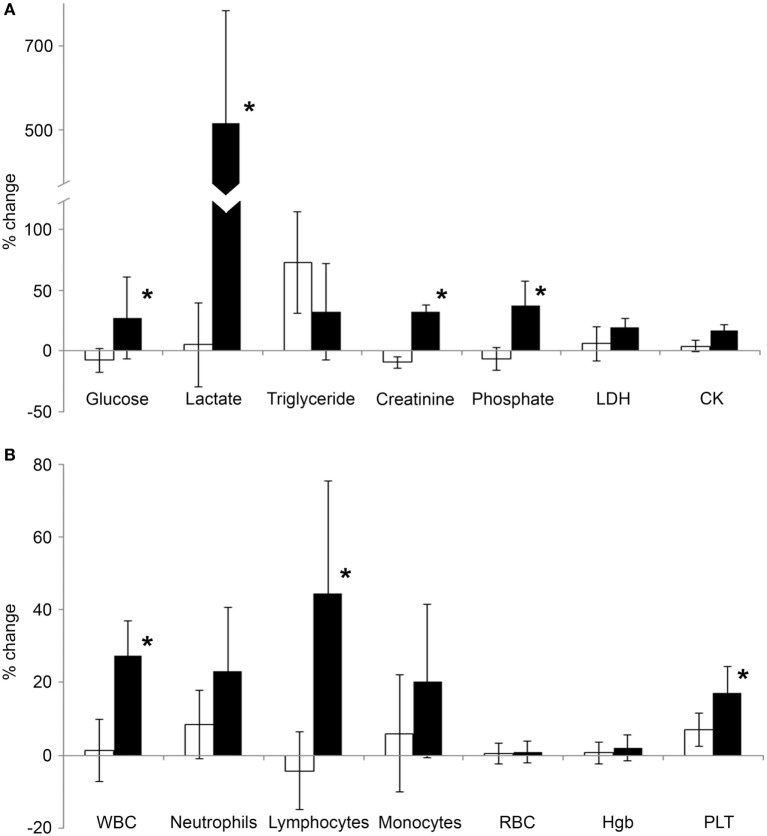
Average change of serum biochemical and blood cell parameters following exercise. Blood was collected from volunteers in the morning, 3 h prior to the exercise, and then a second blood sample was collected from the same individuals after a 2-mile running exercise. Previously, the same experimental protocol was also carried out with the same individuals when they were asked to rest instead of running. **(A)** Average intra-daily changes of various serum biochemical parameters related to metabolism (glucose, lactate, triglyceride, creatinine, and phosphate) or tissue damage [lactate dehydrogenase (LDH), CK] were calculated after exercise (black bars) or after resting (open bars). **(B)** Average intra-daily changes of the key blood cell parameters were calculated in samples collected after exercise (black bars) or after resting (open bars). Relative changes after exercise or resting were expressed as percentages of the corresponding, “before” values. Data are mean ± SD, *p < 0.05 vs the change of the resting condition.

A series of biochemical parameters were analyzed from the blood, including ions, metabolic parameters, tissue damage, and inflammatory markers, and cellular parameters. We found that all parameters were within the normal range when measured from the early morning (before resting and before exercise) samples (Table 2). There was no significant difference in any of the measured parameters when the first samples of the two phases were compared (before resting vs before exercise). In the first phase, when the volunteers were asked to restrain from any physical activity, a few parameters changed significantly in the second (after resting) blood samples: insulin, triglyceride, albumin, and creatinine. These changes albeit statistically significant, could be attributed to diurnal variations (albumin and creatinine) or to the metabolic, post-prandial effect of the standardized breakfast (insulin and triglyceride). However, many of the blood parameters changed following exercise: phosphate, lactate, creatinine, lactate dehydrogenase (LDH), albumin, WBC, lymphocyte, and platelet counts significantly increased when compared to their corresponding parameters measured before exercise. To exclude the influence caused by either diurnal variations or post-prandial effects, we also compared the intra-daily changes in the resting phase with the intra-daily changes in the exercise phase (Figure 1). We have found that exercise compared to resting had a significantly different impact on glucose (average intra-daily change: 27.2 vs −7.9%), lactate (515 vs 5%), creatinine (32 vs −9.6%), phosphate (37.5 vs −6.3%), WBC (27.6 vs 1.3%), lymphocyte (44.5 vs −4.4%), and platelets (17.3 vs 7.1%) values.

### Flow Cytometry Shows Elevated O-GlcNAc in Lymphocytes and Monocytes

**Figure 2:**
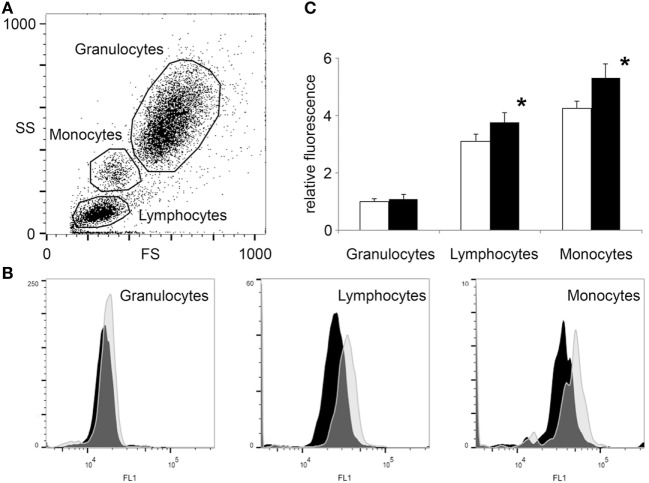
Flow cytometry shows increased O-GlcNAc levels in lymphocytes and monocytes but not in granulocytes after exercise. White blood cells were isolated from blood collected 3 h before and after a 2-mile running exercise. After labeling with anti-O-GlcNAc antibody RL2 and green fluorescent secondary antibody (detected at FL1 channel), the cells were counted by flow cytometry. **(A)** Data points are shown as a function of forward scatter and side scatter (FS—*x*-axis, SS—*y*-axis). Three major group of cells were selected; granulocytes, lymphocytes, and monocytes. **(B)** The O-GlcNAc staining (FL1 channel) of the three regions of cells were individually displayed by histograms. Distribution of cells collected before exercise are presented by black histograms, while the distribution of cells collected after exercise are presented by light gray histograms. **(C)** Relative FL1 fluorescence levels of O-GlcNAc labeled blood cells, collected before (open bars) and after (black bars) exercise. The mean fluorescence level of the granulocytes collected before exercise was selected for baseline. Data are shown as mean ± SD, *p < 0.05 vs before exercise.

To assess the effect of physical exercise on WBCs, EDTA anti-coagulated blood was collected before and after exercise carrying out the same experimental conditions as described above. Isolated and fixed blood cells were fluorescently labeled for O-GlcNAc with RL2 anti-O-GlcNAc antibody. Figure 2 shows that based on the forward scatter and side scatter values [generally accepted to report cell size and granularity (39)], three distinct group of cells could be clearly identified; granulocytes, monocytes, and lymphocytes. Restricting the selection of the cells to the regions highlighted in Figure 2A, O-GlcNAc levels (FL1 fluorescence) of the three cell types were analyzed separately. We found that granulocytes had approximately the same O-GlcNAc levels before and after exercise (relative fluorescence: 1.0 ± 0.1 vs 1.1 ± 0.16, NS), while lymphocytes (relative fluorescence: 3.1 ± 0.26 vs 3.8 ± 0.34, *p* < 0.05) and monocytes (relative fluorescence: 4.2 ± 0.25 vs 5.3 ± 0.48, *p* < 0.05) showed a significant right shift (i.e., increased level of O-GlcNAc) after physical exercise (Figures 2B,C).

Elevation of O-GlcNAc levels in a population of cells could be either the result of an overall increase in every cell, or a larger increase in a subpopulation of the cells. In our study, lymphocyte count increased following exercise (2.3 ± 0.5 vs 3.2 ± 0.8 G/L, *p* < 0.05, Table 2), suggesting that a new subset of cells carrying more O-GlcNAc might have entered the circulation (40). The distribution of cells in the FL1 histogram could resolve whether O-GlcNAc elevation is due to an elevation of O-GlcNAc across all lymphocytes or due to the appearance of a new, above than average O-GlcNAcylated subset of cells. Our experiments support the former case; we found that lymphocytes showed near Gaussian distribution both before and after exercise (Figure 2B). Although the analysis of monocyte distribution is much less reliable due to their lower proportion (0.4 ± 0.1 G/L before and 0.5 ± 0.1 G/L after exercise, NS), monocytes appeared to have at least two types of subpopulation when O-GlcNAc levels were analyzed. Since we found no significant change in monocyte count after exercise, it is possible that a subset of monocytes is more sensitive to exercise than the rest.

### Elevation of O-GlcNAc in Mononuclear Cells After Exercise Confirmed by Western Blot

**Figure 3:**
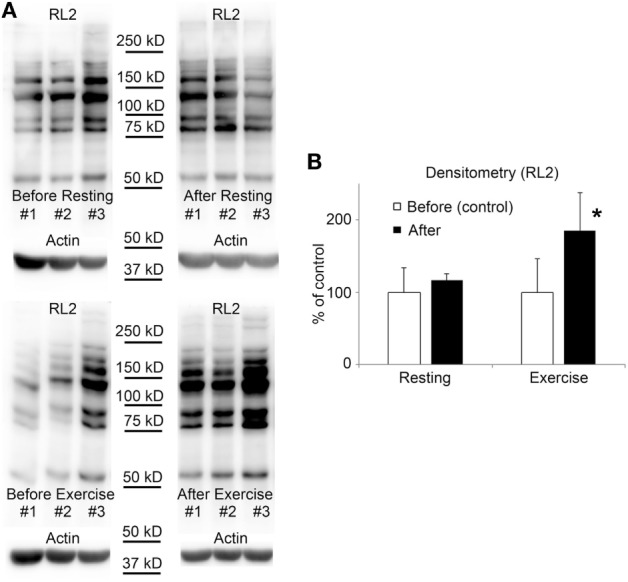
O-GlcNAc levels elevate in leukocytes following exercise. **(A)** Western blot analysis using RL2 antibody and anti-actin antibody shows samples of white blood cells protein extracts from three individuals. Sample were collected 3 h before exercise and before resting (left panels), and also after exercise and after resting (right panels). **(B)** Densitometry analysis of the RL2 staining is expressed as a percentage of the control samples (i.e., samples collected before exercise or before resting). Each data point shows the average of six individual samples. Open bars represent the mean ± SD of “before” samples and black bars represent the mean ± SD of “after” samples. *p < 0.05 vs before exercise.

Blood samples were collected parallel with the samples used for testing the biochemical parameters; before/after resting and exercise. Mononuclear cells were isolated from EDTA anti-coagulated blood by Histopaque-1077 separation immediately after blood collection. Figure 3 shows the level of O-GlcNAc proteins of three volunteers, before and after physical exercise or resting. The resulting banding patterns were similar to those published in previous reports (41, 42). As expected, we have found no significant changes in O-GlcNAc levels when the volunteers were asked to rest between blood collections. However, an approximately 15 min of intensive running caused a significant increase in intensity of overall O-GlcNAc staining (85 ± 51% increase, *p* < 0.05) when compared to the samples collected before exercise (16 ± 9% increase, NS).

**Figure 4:**
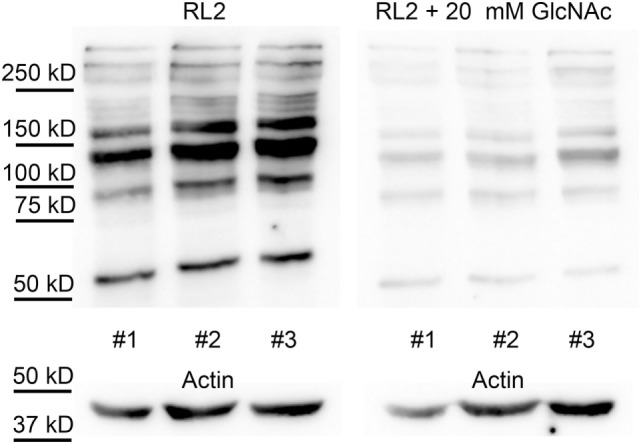
RL2 specifically binds to O-GlcNAc proteins. Western blot analysis using RL2 antibody and anti-actin antibody shows samples of white blood cells protein extracts from three individuals, collected at random dates. Membranes were incubated with RL2 antibody (left panel) or RL2 antibody and 20 mM of *N*-acetylglucosamine (right panel). Immunoblots with anti-actin antibody was used as loading control.

To demonstrate the specificity of the O-GlcNAc antibody, a duplicate immunoblot was prepared by co-incubating RL2 antibody with 20 mM *N*-acetylglucosamine. As shown in Figure 4, *N*-acetylglucosamine decreased the western blot signal by about five times, compared to the uninhibited samples (19 ± 2% of control).

## Discussion

In the present study, we have analyzed the effect of a 2-mile running exercise on the level of O-GlcNAc modification in leukocytes collected from human volunteers. We have found that overall level of O-GlcNAc modified proteins significantly increased following exercise when compared to control, resting experimental conditions. We have also found that lymphocytes and—to a lesser extent—monocytes were mostly responsible for this increase while the O-GlcNAc levels of granulocytes remained relatively unchanged. Our data also suggest that the elevation of O-GlcNAc is a general event rather than a redistribution of certain lymphocyte subpopulations (carrying various amount of O-GlcNAc modification) in the circulation.

Our study was designed to focus on a single bout of physical challenge and to study the acute effects of exercise on O-GlcNAc levels in leukocytes. The selection of the participants (healthy, young, and trained individuals) and the duration of the physical activity ensured that the workload does not exceed the adaptation capacity of the participants. Biochemical markers sensitive for tissue damage (LDH, CK)—although elevated—remained close to normal range. IL-6 levels—which are known to increase proportional to the amount of exercise—remained below 2 ng/L (data not shown). Lactate levels after exercise (6.2 ± 2.0 mM) showed that the workload corresponded to approximately the state where aerobic–anaerobic transition occurs (~4 mM lactate) (43). Elevated level of serum phosphate and increased number of circulating leukocytes were expected results after exercise and are in good correspondence with the literature (44–46). In our study, only male volunteers were recruited to avoid interference due to hormonal differences between sexes. However, the influence of gender or cyclic hormonal changes on O-GlcNAc regulation needs to be studied in the future. The relatively few data available in the literature indeed suggest that O-GlcNAc interacts with hormonal receptors (46) including estrogen receptor (47, 48). It is also interesting to note that one of the regulatory enzymes of O-GlcNAc; O-GlcNAc transferase (OGT) is X-linked. Without X-inactivation, the expressional level of OGT would be approximately double in female cells (49).

Increasing amount of data demonstrates that the immune system is also influenced by physical activity, and moreover it is part of the systemic adaptation and response to physical challenges (50). Demargination of leukocytes and leukocytosis was recognized early as a consequence of physical exercise (45, 51). Limited inflammatory response can be elicited during exercise by muscle micro-injuries, bacterial translocation from the gut due to reduced blood flow or due to direct oxidative stress of various elements of the immune system (7, 37). On the other hand, activated leukocytes and inflammatory response consequently supports healing and regeneration (37, 52). There are many factors that may influence the actual outcome of the immunomodulatory effect of exercise the length and intensity of the exercise, whether it is aerobic or anaerobic and also the presence or absence of previous training exercise (53, 54). “Overreaching,” i.e., performing more strenuous exercise than the body can tolerate will lead to immunosuppression while moderate exercise will actually benefit the immune system and reduce infection incidence (50, 55). The distinction between pro- and anti-inflammatory effects of exercise is not clearly defined but it is rather a dynamic balance and the actual result is a mixture of a multitude of signaling elements (53).

As mentioned, protein O-GlcNAc modification has been proposed as a protective intracellular mechanism against various type of stress, including hypoxia, oxidative stress, heat-shock, and osmotic challenges (22). It was demonstrated previously (31, 56, 57) and we have also found in our present experiments that O-GlcNAc can elevate within minutes. Stress-induced O-GlcNAc may have several consequences: it may influence transcription (e.g., increasing the expression of heat-shock proteins), phosphorylation signaling, or protein degradation (18, 58). Immediate effects of O-GlcNAc that do not require *de novo* protein synthesis include the inhibition of enzymes involved in oxidative stress such as nitric oxide synthase (59, 60). Finally, it seems to be that O-GlcNAc may also suppress stress-induced intracellular free calcium elevation, which would otherwise lead to adverse effects (57). In our present report, we propose that O-GlcNAc modification may regulate immune response during exercise as part of the stress response system.

Several studies found that O-GlcNAc may participate in inflammatory responses (61–66), but the exact function and role of O-GlcNAc in the regulation of inflammation is still unclear (67, 68). For example, one of the key signal elements of the immune response, NFκB was shown not only to be activated by O-GlcNAc (41, 66, 69) but also to be inhibited by O-GlcNAc (64, 65, 70). This contradiction of results is probably caused by a complex interplay between several O-GlcNAc and phosphorylation sites on NFκB. Depending on the cell type, the duration, type and severity of stress, different posttranslational patterns may develop on NFκB and produce different outcomes. Despite uncertainties concerning the function of O-GlcNAc modification in immune cells, accumulating data suggest that O-GlcNAc elevation in WBCs is a quick and measurable response to activation (32, 71).

Our present work is the first that shows an elevation of O-GlcNAc levels in human WBCs following acute stress, i.e., physical exercise. Only a few studies used human WBCs to measure O-GlcNAc levels yet; in these studies, western blot and flow cytometry techniques were also used to demonstrate that O-GlcNAc levels can be assessed in human leukocytes (33, 35, 42). In contrast to western blot, flow cytometry may also allow for comparison of various subsets of leukocytes. As of now, flow cytometric studies, including our present work have only a limited resolution of leukocyte sub-types (granulocytes, lymphocytes, and monocytes) based on forward and side scatter plots. Thus our results have to be confirmed in the future by the simultaneous labeling of O-GlcNAc proteins and specific leukocyte surface markers such as CD45, CD3, CD19, CD4, CD8, etc.

A study by Myslicki et al. used human whole blood samples to measure O-GlcNAc (34). They found no correlation between whole blood O-GlcNAc and aerobic capacity (VO_2peak_) in healthy males, however, their samples were collected only from resting individuals. The direct activator of O-GlcNAc elevation in our experimental setup is not known yet. Several physical and biochemical factors could be responsible, such as acidosis, relative hypoxia, oxidative stress, increase of temperature, myokin release from the muscles, hormonal and metabolic changes (epinephrine, norepinephrine and glucose, insulin), or increased hemodynamic shear stress due to faster circulation. Our preliminary data on a Jurkat (T-cell derived) cell culture model suggest that lactic acid alone (up to 10 mM) is not sufficient to elicit a significant increase of O-GlcNAc (data not shown). Agonist, such as epinephrine and angiotensin II (57) and/or oxidative stress (72) are also likely candidates responsible for the O-GlcNAc elevation observed in our study but so far there is no experimental evidence available in leukocytes or leukocyte-derived cells to confirm it.

Under moderate-intensity exercise, blood glucose levels should remain relatively stable; while the muscles’ glucose uptake is increased during exercise, it is balanced by increased glucose production by the liver (73). Nevertheless, fluctuations in blood glucose levels could be a plausible explanation for the measured changes in O-GlcNAc levels. Hypoglycemia are known to cause a paradoxical increase in O-GlcNAcylation (74), however, it is unlikely that the duration of the exercise in our experimental setup would be sufficient to decrease glucose levels. On the contrary, the measured mean plasma glucose values—despite large individual variations—seemed to be slightly increased. Unfortunately, most studies investigated the effect of hyperglycemia with much higher glucose concentrations (25–30 mM). Moderately elevated glucose levels (~1 mM increase) were shown to increase O-GlcNAc levels only under long-term conditions (pre-diabetes) so far (35, 75).

In summary, our study demonstrated for the first time that following a single bout of running exercise, protein O-GlcNAc modification increased in WBCs. We have also shown that the bulk of this change is attributed to lymphocytes, while granulocytes remained relatively unchanged. While the understanding of the complex function of O-GlcNAc modification in leukocytes requires further extensive studies, we believe that our results presented here will help to elucidate the role of O-GlcNAc in stress adaptation mechanisms. Implications could include the development of new diagnostic tools for the monitoring of healthy (sport medicine) and metabolically unhealthy (e.g., diabetes) population as well.

## Ethics Statement

This study was carried out in accordance with the recommendations of Guidelines of the Regional Committee for Research Ethics of the University of Pécs, Hungary. All subjects gave written informed consent in accordance with the Declaration of Helsinki. The protocol was approved by the Regional Committee for Research Ethics of the University of Pécs, Hungary (approval No.: 5187).

## Author Contributions

Study design and organization of the manuscript were perfor-med by TN, AS, IW, and AM. Data analysis, statistical analysis, and the first draft of the manuscript were performed by TN, EK, VF, TT, and AS. The manuscript review was performed by EK, VF, and TT. The final approval for publication was performed by TN, IW, and AM.

## Conflict of Interest Statement

The authors declare that the research was conducted in the absence of any commercial or financial relationships that could be construed as a potential conflict of interest.
